# Population structure of *Bactrocera dorsalis s.s.*, *B. papayae* and *B. philippinensis* (Diptera: Tephritidae) in southeast Asia: evidence for a single species hypothesis using mitochondrial DNA and wing-shape data

**DOI:** 10.1186/1471-2148-12-130

**Published:** 2012-07-30

**Authors:** Mark K Schutze, Matthew N Krosch, Karen F Armstrong, Toni A Chapman, Anna Englezou, Anastasija Chomič, Stephen L Cameron, Deborah Hailstones, Anthony R Clarke

**Affiliations:** 1 CRC for National Plant Biosecurity, LPO Box 5012, Bruce, 2617, A.C.T, Australia; 2 School of Earth, Environmental and Biological Sciences, Queensland University of Technology, G.P.O. Box 2434, Brisbane, 4000, QLD, Australia; 3 Bio-Protection Research Centre, Lincoln University, PO Box 84, Lincoln, 7647, Christchurch, New Zealand; 4 NSW Department of Primary Industries, Elizabeth Macarthur Agricultural Institute, Woodbridge Rd, Menangle, 2568, NSW, Australia

**Keywords:** Geometric morphometrics, Cytochrome *c* oxidase I, South China Sea biogeography, Fruit flies

## Abstract

**Background:**

*Bactrocera dorsalis s.s.* is a pestiferous tephritid fruit fly distributed from Pakistan to the Pacific, with the Thai/Malay peninsula its southern limit. Sister pest taxa, *B. papayae* and *B. philippinensis*, occur in the southeast Asian archipelago and the Philippines, respectively. The relationship among these species is unclear due to their high molecular and morphological similarity. This study analysed population structure of these three species within a southeast Asian biogeographical context to assess potential dispersal patterns and the validity of their current taxonomic status.

**Results:**

Geometric morphometric results generated from 15 landmarks for wings of 169 flies revealed significant differences in wing shape between almost all sites following canonical variate analysis. For the combined data set there was a greater isolation-by-distance (IBD) effect under a ‘non-Euclidean’ scenario which used geographical distances within a biogeographical ‘Sundaland context’ (*r*^*2*^ = 0.772, *P* < 0.0001) as compared to a ‘Euclidean’ scenario for which direct geographic distances between sample sites was used (*r*^*2*^ = 0.217, *P* < 0.01). COI sequence data were obtained for 156 individuals and yielded 83 unique haplotypes with no correlation to current taxonomic designations via a minimum spanning network. beast analysis provided a root age and location of 540kya in northern Thailand, with migration of *B. dorsalis s.l.* into Malaysia 470kya and Sumatra 270kya. Two migration events into the Philippines are inferred. Sequence data revealed a weak but significant IBD effect under the ‘non-Euclidean’ scenario (*r*^*2*^ = 0.110, *P* < 0.05), with no historical migration evident between Taiwan and the Philippines. Results are consistent with those expected at the intra-specific level.

**Conclusions:**

*Bactrocera dorsalis s.s.*, *B. papayae* and *B. philippinensis* likely represent one species structured around the South China Sea, having migrated from northern Thailand into the southeast Asian archipelago and across into the Philippines. No migration is apparent between the Philippines and Taiwan. This information has implications for quarantine, trade and pest management.

## Background

**Figure 1:**
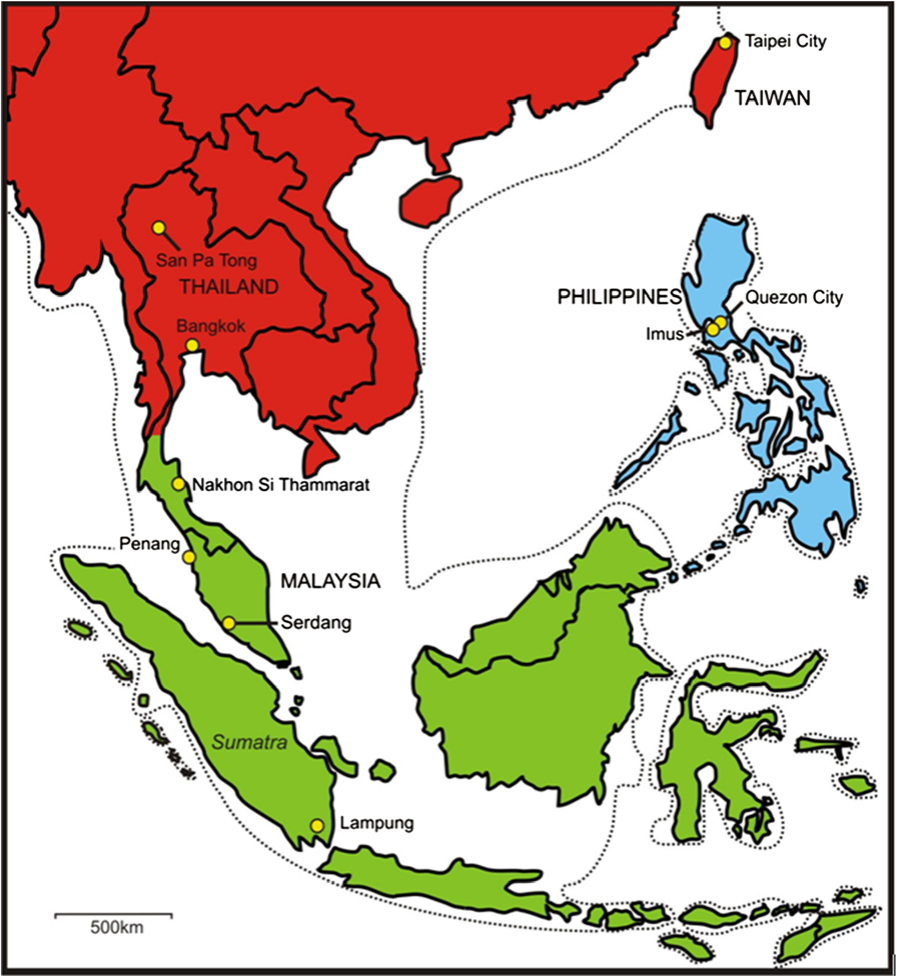
**Sample sites of* B. dorsalis s.l. *in southeast Asia.** Map of southeast Asia showing sample sites from which *Bactrocera dorsalis* complex flies were collected for this study. Different coloured regions denote the generally accepted geographic distributions and inferred zones of transition among *B. dorsalis s.s.* (red), *B. papayae* (green) and *B. philippinensis* (blue; but also recorded from Borneo) based on [4] and [80]. For illustrative purposes, the dotted line represents the approximate coastline for when sea levels dropped to 120 m below current levels exposing the Sunda shelf and forming the extensive region of ‘Sundaland’ (redrawn from [28]).

The *Bactrocera dorsalis* species complex (Diptera: Tephritidae) is a group of over 70 fruit fly species, some of which are major horticultural pests [1]. Among the most damaging species within the complex are *B. dorsalis s.s.B. papayae* and *B. philippinensis* as they attack a wide variety of fruit, have high reproductive potential, and all three are known invasives [2,3]. These three species occur across broad but essentially allopatric distributions; with regions of transition occurring around the South China Sea in southeast Asia (Figure 1) [4]. Research and management of these species is confounded by their close morphological [1,4,5], genetic [6-9], physiological [10] and behavioural similarities [11,12]. *Bactrocera papayae* and *B. philippinensis* were erected as new species separate from *B. dorsalis s.s.* in 1994 following a revision of the complex [4]. However they were separated based on subtle morphological characters: *B. papayae* was distinguishable from *B. dorsalis s.s.* in having a longer aculeus and was deemed separate from *B. philippinensis* based on morphological variation in the scales on the distal end of the middle segment of the aculeus; and while *B. philippinensis* was recognised as ‘*difficult to separate’* from *B. dorsalis s.s.* it was considered a new species based on differences in the mean ratio of wing vein lengths (CuA1 along dm cell) to the length of the aculeus (1.19 in *B. philippinensis* and 1.47 in *B. dorsalis s.s.*) [4]. These characters are extremely difficult to apply to practical diagnoses of adult specimens (let alone immature samples) and is further compounded by these characters being female-specific, a real operational problem as the most commonly used traps for these species are methyl-eugenol baited and attract only males [1]. Consequently, the identification of these species relies heavily on their respective geographical distributions to discriminate amongst them [4], despite known problems in using geography as a taxonomic character [13-15]. Thus in contrast to the three taxa representing unique biological species structured around the South China Sea, an equally parsimonious hypothesis is that they are a single widely distributed species for which subtle differences represent variation at the intra- rather than interspecific level.

Population studies on the *B. dorsalis* species complex have focused on *B. dorsalis s.s.* within that species’ described geographic distribution, predominantly northern southeast Asia and southern China, west to Bangladesh and east to the Pacific [16-21]. Current theory based on genetic data places the origin of *B. dorsalis s.s.* either in southern-central China [16], or the southeast corner of mainland China [22]. Genetically diverse populations in southern China are as distinct from each other as from those in southeast Asia (i.e., Myanmar, Laos, and Vietnam) and are believed to be structured by mountain ranges and air currents, rather than purely by geographic distance [17,18,21]. Dispersal of *B. dorsalis s.s.* individuals is generally limited to within 50 km of their origin [23-25]; however longer distance fly movements (presumably wind assisted) of 100 – 250 km have been reported in southern China [19,26]. A combined analysis of population structure of *B. dorsalis s.s.* with its closely related sibling species, *B. papayae* and *B. philippinensis*, in the biogeographically complex South China Sea area has never been undertaken. This is primarily because the three taxa are regarded as distinct species, each occupying different distributions within the region: *B. dorsalis s.s.* around the northern edge (north of the Thai/Malay peninsula), *B. papayae* around the western and southern edge (Thai/Malay peninsula and into the Indonesian archipelago) and *B. philippinensis* at the eastern edge (the Philippines and Borneo) (Figure 1). However if no *a priori* assumptions are made that these taxa represent separate species then a population-level analysis using samples obtained throughout this region may contribute towards resolving species boundaries. And given the geographical complexity of southeast Asia around the South China Sea, such an analysis should be undertaken in the context of the region’s history.

The region of southeast Asia surrounding the South China Sea has experienced considerable tectonic activity [27], repeated sea level fluctuations [28] and associated climatic and vegetation changes [29,30]. Numerous cycles of sea level changes over the last 250,000 years [28], including several periods during which sea levels fell to 120 m below present, resulted in repeated connections between today’s mainland southeast Asia and the major islands of Sumatra, Java, and Borneo via the Thai-Malay peninsula. Such sea level drops exposed the vast Sunda Shelf and formed the region known as ‘Sundaland’ [28,31]. During periods when Sundaland was exposed to its maximum area (the size of Europe, see Figure 1), the landmass was vegetated by an essentially open savannah corridor interspersed by forest refugia [29]. This allowed increased migration of fauna, including forest-dependant species, throughout the present island chains of the region [29]. However as sea levels rose, the Sunda Shelf re-submerged and land-bridges – such as the Thai-Malay Isthmus of Kra – narrowed. Furthermore, closed forest habitats throughout the region were periodically reduced to isolated refugia in response to cooling climates [30]. These events enforced dispersal barriers, restricting movement from mainland southeast Asia into the peninsula and archipelago for many species of invertebrates, reptiles, birds and mammals [32-37]. Given this complex history, a unified biogeographic pattern for all local taxa is unlikely [38], and any biogeographic study within this region must be examined on its own merits. Regarding *B. dorsalis s.l.*, for example, we may predict that the historical movement of flies throughout this region may have been facilitated during the exposure of Sundaland, and subsequently restricted as sea levels once again rose to potentially cut off dispersal routes.

We have identified the need to study the genetic and morphological variation of *B. dorsalis s.l.* in an area which had not previously been investigated, i.e., extending southwards of mainland China and including the region surrounding the South China Sea. In light of the possibility that *B. dorsalis s.s.B. papayae* and *B. philippinensis* represent the same biological species (further supported by the authors’ unpublished work demonstrating mating compatibility), we approached this study with no *a priori* assumptions based on current taxonomic designations. If these three species represent one biological entity, we predict to observe active gene flow among the groups alongside results consistent with prior population genetic studies depicting a south Chinese origin; the latter being in line with expectations based on a biogeographical history of migration southwards from China into the Indonesian archipelago and across to the Philippines. Alternatively, if the current taxonomy of the three species is correct, we predict very different results. These include tighter correlations between taxonomic species designations and haplotype distributional relationships, consistent and significant differences in pair-wise measures of genetic or morphometric distances between taxonomic species but not for populations within them, and poor isolation by distance effects across the breadth of the sampled geographic range. Furthermore, we have explicitly chosen to exclude the closely related species *B. carambolae* Drew & Hancock from comprehensive analysis presented here. While this species is closely allied to *B. dorsalis s.s.B. papayae* and *B. philippinensis*[8,9], a comprehensive survey of *B. carambolae* across its native and invasive ranges has revealed it to i) be reciprocally monophyletic (via a muli-locus phylogenetic analysis; authors’ unpublished data); ii) relatively sexually incompatible (authors’ unpublished data); and iii) not share any COI haplotypes with the latter three species (see Methods and Additional file 1); thereby confirming its specific status as separate from *B. dorsalis s.s.B. papayae* and *B. philippinensis* and warranting its exclusion from the population-level analyses presented here.

For this study, the cytochrome *c* oxidase subunit 1 (COI) mitochondrial DNA (mtDNA) gene was selected for analysis as it is a relatively fast evolving and practical locus from which to derive recent evolutionary genetic histories. The COI gene has also been successfully applied previously to assess population structure for *B. dorsalis s.s.*[17-20] and for a diverse variety of other insects [39-44]. Although we are aware of the caveats regarding the use and interpretation of mtDNA in studies of historical population structure [45,46], especially where the focus is the species and not the organelle [47], its usage in conjunction with other methods is still generally considered a valid contribution towards assessing species divergence and historical population characteristics. In addition to mtDNA analysis, we therefore apply geometric morphometric shape analysis of wings as an independent measure of population structure for *B. dorsalis s.s.B. papayae* and *B. philippinensis*. Geometric morphometric analysis quantifies shape variation among individuals or defined groups [48,49], and such information has demonstrated effectiveness for discriminating groups at both inter- and intraspecific levels across a range of taxa [50-53], including members of the *B. dorsalis* species complex [54]. While the use of geometric morphometric shape analysis in studies of population structure continues to grow, its direct use with genetic data from the same individuals as an independent and parallel dataset remains uncommon despite its documented potential [51,55,56].

This study therefore uses two independent data sets (mtDNA and wing shape) to assess geographic variation for flies sampled across much of the distributional range of *B. dorsalis s.s.*, *B. papayae,* and *B. philippinensis* in order to assess whether such variation aligns with that expected at the intra- or interspecific level. We further aimed to determine if the historical migration patterns hypothesised from our data concur with what is known of the regions’ rich geographical history.

## Results

### Geometric morphometrics

One hundred and sixty nine individuals taxonomically identified as *B. dorsalis s.s., B. papayae* or *B. philippinensis* were collected from nine sites and used for geometric morphometric analysis (Table 1). Right wings were used for the majority of samples (~90%) and each had 15 landmarks digitised for analysis. Analysis of variance of centroid sizes among groups was statistically significant (*F*_(8,160)_ = 4.772; *P* < 0.001), however the Tukey *post hoc* test revealed no particular trend for variation in centroid size associated with location or species (see Additional file 1: Figure S1). The largest wings, on average, belonged to individuals sampled from Imus, Philippines (mean centroid size 6.60 ± 0.10 s.e.) and the smallest to those from Penang, Malaysia (5.96 ± 0.07).

**Table 1:** **Collection and sample size data for* B. dorsalis s.l. *in this study**

**Sample site**	**Taxonomic species**	**Latitude**	**Longitude**	**Sample size (genetics/shape)**	**Number of COI haplotypes**
Taipei, China (Taiwan)	*B. dorsalis s.s.*	25°00'53"N	121°32'18"E	19/20^1^	16
San Pa Tong, Thailand	*B. dorsalis s.s.*	18°37'37''N	98°53'42''E	19/20^0^	16
Bangkok, Thailand	*B. dorsalis s.s.*	13°50'32"N	100°34'23"E	19/20^1^	18
Nakhon Si Thammarat, Thailand	*B. dorsalis s.s.*	8°25'12''N	99°53'48''E	12/20^2^	10
Penang, Malaysia	*B. papayae*	5°28'33''N	100°17'51''E	20/20^2^	17
Serdang, Malaysia	*B. papayae*	3°00'20''N	101°42'00''E	22/20^4^	11
Lampung, Indonesia	*B. papayae*	5°40'43''S	105°36'38''E	15/14^0^	5
Quezon City, Philippines	*B. philippinensis*	14°38'00''N	121°01'00''E	17/20^5^	2
Imus, Philippines	*B. philippinensis*	14°07'18"N	120°58'00"E	13/15^2^	5

Geographical location of sample sites for *Bactrocera dorsalis* complex flies used in this study, number of individuals from each sample site used for genetic and geometric morphometric shape analysis, and number of COI haplotypes identified for each sample site. Superscript values for sample sizes used in geometric morphometric shape analysis are the number of left-hand wings used.

**Figure 2:**
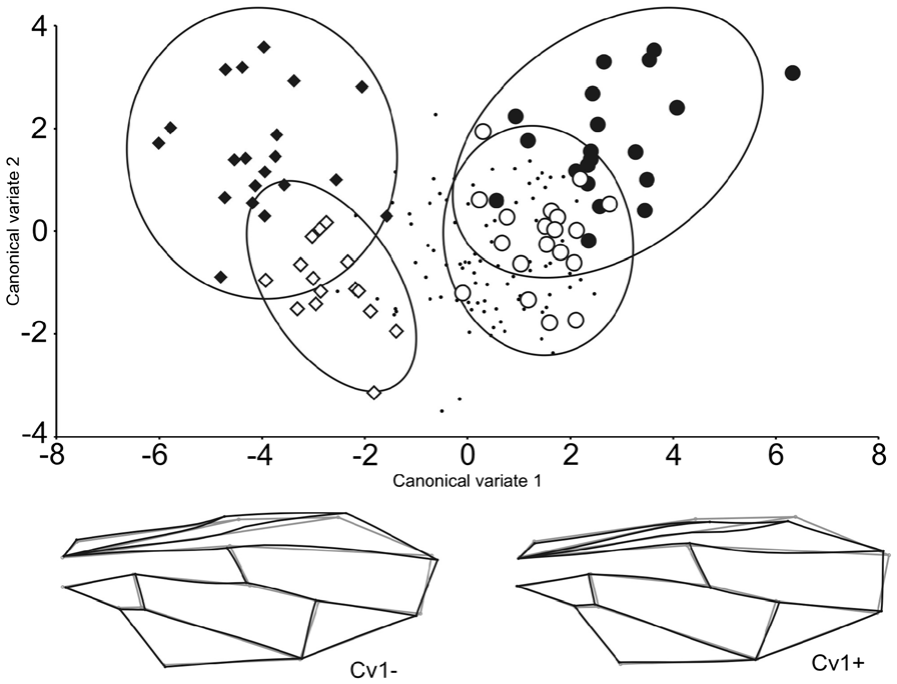
**First two canonical variates following* B. dorsalis s.l. *wing shape analysis.** Plot of wing shape data from * B. dorsalis * complex flies along first two canonical variates following CVA. For clarity, only four sites are highlighted with 95% confidence ellipses: diamonds = Philippines (closed = Quezon City; open = Imus); open circles = San Pa Tong, nth Thailand; filled circles = Taiwan, China. Remaining sample sites indistinguishable in this plot and are represented as small dots. Black wireframe wing images denote shape deformation along the first canonical variate in the positive (Cv1+) and negative (Cv1-) direction (scale factor 10); grey wireframe = consensus shape.

The regression of the shape variable on centroid size was statistically significant (*P* < 0.0001) and accounted for 4.71% of shape variation. Consequently further analyses were conducted on data corrected to account for allometric effect. Canonical variate analysis based on the nine sample locations revealed eight canonical variates of which the first two accounted for 70% of the variation (see Additional file 1: Table S1). Based on the first two canonical variates, there is separation of the Philippine populations (Quezon City and Imus) from groups sourced from the remaining sample sites, with the greatest difference occurring along CV1 between both Philippine sites and Taiwan (Figure 2). Shape deformations along CV1 are principally represented by landmarks 12, 14 and 15 moving distally with respect to other landmarks as CV1 decreases, coupled with slight shifts in cross-vein configurations (Figure 2). Remaining sites are largely indistinguishable along these first two CVs; however they are further resolved from each other along other CVA dimensions as revealed by comparisons among group Mahalanobis distances (see Additional file 1: Table S2).

*A priori* groups were significantly different (*P* < 0.05) from each other based on permutation tests for Mahalanobis distances for all comparisons except that between San Pa Tong (north Thailand) and Bangkok (central Thailand) (*P* = 0.41) (see Additional file 1: Table S2). Comparisons of Procrustes distances yielded fewer significant differences among sample sites. Sites located on the Thai/Malay peninsula and Sumatra (i.e. San Pa Tong, Bangkok, Nakhon Si Thammarat, Penang, Serdang, and Lampung) were not significantly different from each other with respect to Procrustes distances, whereas Taiwan and both Philippine sites (Quezon City and Imus) were significantly different for all pairwise comparisons, including the notable difference between Quezon City and Imus despite their geographic proximity (see Additional file 1: Table S2).

**Figure 3:**
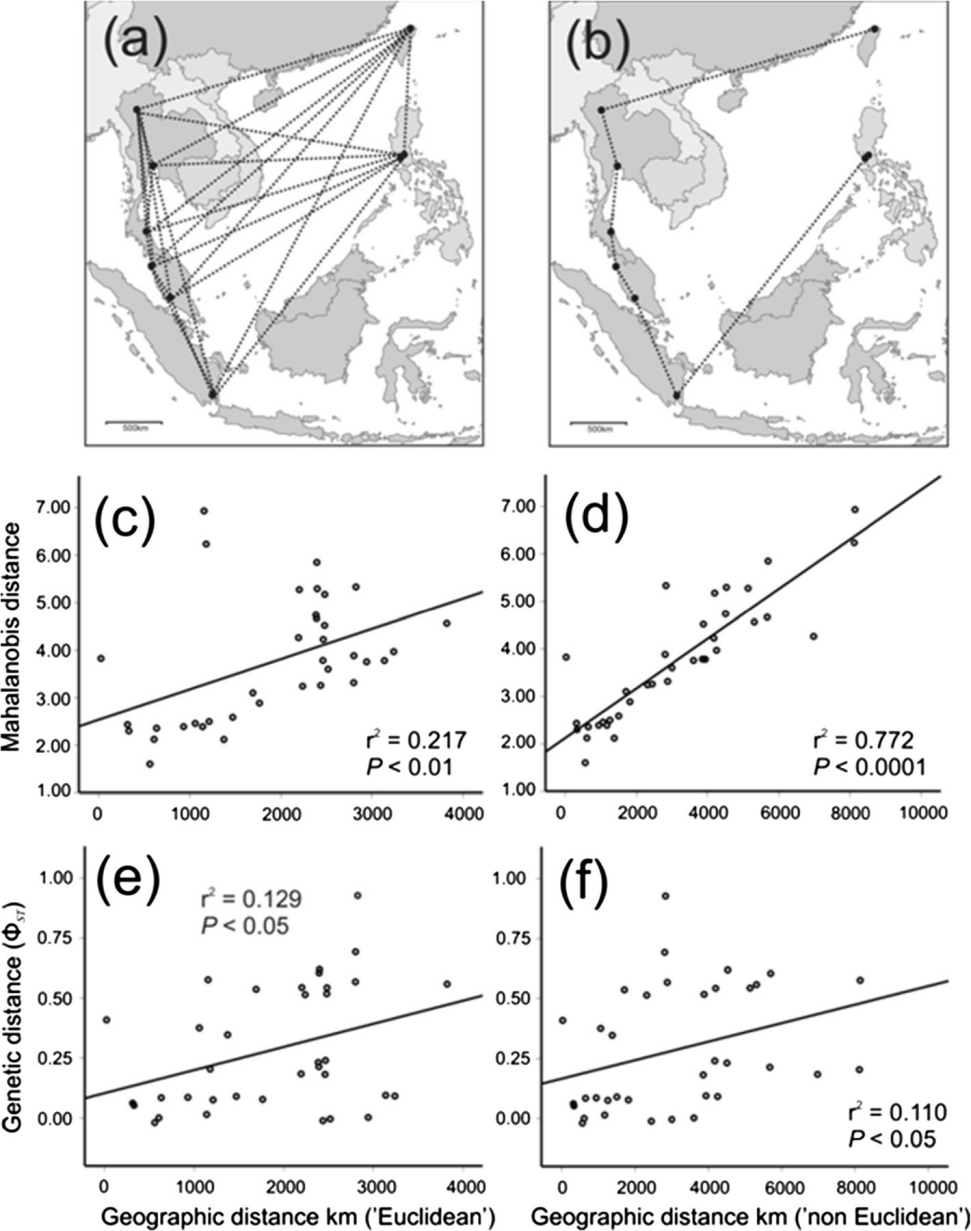
**Linear regression analysis of genetic and wing shape data for* B. dorsalis s.l. *against geographic distances between sample sites.** Linear regression analysis of genetic (pairwise ΦST) and Mahalanobis distances (from CVA) among groups against geographic distances between sample sites (km) for both the ‘Euclidean’ (left) and ‘non-Euclidean’ (right) comparisons. (**a**) = Map of southeast Asia region showing Euclidean geographic distance measures; (**b**) = Map of region showing ‘non-Euclidean’ geographic distances; (**c**) = Mahalanobis distance vs Euclidean distance; (**d**) = Mahalanobis distances vs ‘non-Euclidean’ distance; (**e**) = Genetic distance vs Euclidean distance; (**f**) = Genetic distance vs ‘non-Euclidean’ distance.

The regression of geographic distance against Mahalanobis distances among groups using Euclidean distances between sample sites (i.e. shortest possible distances) yielded a significant relationship (Pearson correlation = 0.465; *r*^*2*^ = 0.217; *P* < 0.01) (Figure 3c). However, the analysis using distances between locations that follow the geography about the south China sea (i.e*.* the ‘non-Euclidean’ analysis) yielded a much stronger and highly significant association (Pearson correlation = 0.878; *r*^*2*^ = 0.772; *P* < 0.0001) (Figure 3d).

### Genetic results

Sequence data for the mtDNA gene COI yielded 83 unique haplotypes from 156 individuals from nine sites across southeast Asia (Table 1). The ratio of transitions to transversions was high at 9.108, which could be indicative of sequence saturation or recent divergence, among other scenarios [57]. Measures of genetic diversity within sites suggested that populations were quite diverse (see Additional file 1: Table S3). The population parameter θ_π_ ranged from 0.118 ± 0.218 (Quezon City) to 4.632 ± 2.635 (Serdang) and gene diversity ranged from 0.118 ± 0.101 (Quezon City) to 0.994 ± 0.019 (Bangkok). Indeed, all but three sites (Quezon City, Imus and Lampung) possessed gene diversities greater than 0.9. Tajima’s D tests of neutrality for the total dataset were negative and statistically significant (D = −2.265, *P* < 0.0001), suggesting either that the sequences may be under selection or that populations may have experienced relatively recent historical expansion.

Estimates of pairwise Φ_*ST*_ indicate distinct population differentiation among almost all sites, except among sites within the described geographic range of *B. dorsalis s.s.* (Taiwan, San Pa Tong, Bangkok and Nakhon Si Thammarat); pairwise comparisons among these four sites were non-significant (see Additional file 1: Table S4). In contrast, sites within the described ranges of *B. papayae* and *B. philippinensis* all differed significantly to each other, despite some being geographically proximate (as for the geometric morphometric shape analysis, e.g., Quezon City and Imus).

Regression of genetic distance (pairwise Φ_*ST*_) against geographic distance revealed a weak but significant positive association for both Euclidean (Figure 3e) and ‘non-Euclidean’ geographic distance comparisons (Figure 3f). However, unlike the geometric morphometric results, the regression of genetic distance against Euclidean geographic distance was slightly stronger (Pearson correlation = 0.360; *r*^2^ = 0.129; *P* < 0.05) as compared with the ‘non-Euclidean’ comparison (Pearson correlation = 0.331; *r*^2^ = 0.110; *P* < 0.05).

**Figure 4:**
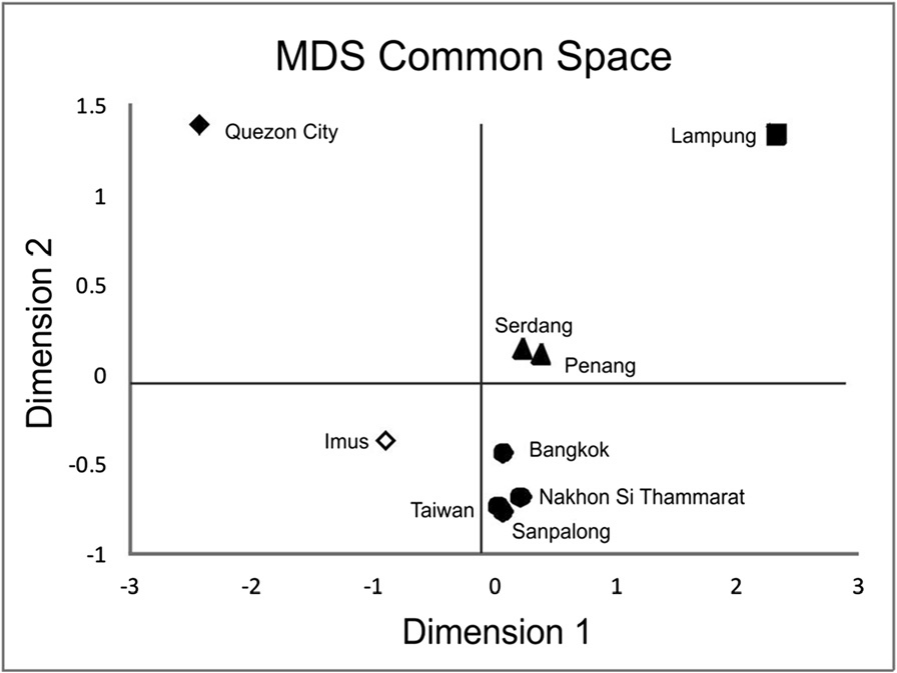
**Multi-dimensional scaling plot based on genetic data of* B. dorsalis s.l. *** Multi-Dimensional Scaling (MDS) plot based on genetic among site ΦST values. Clustering of sites post hoc gave five putative groups, referred to hereafter as: Taiwan/Thailand (filled circles); Peninsular Malaysia (filled triangles); Sumatra (filled square); Philippines Clade A (filled diamond); and Philippines Clade B (open diamond).

Multidimensional scaling plots clustered Taiwanese and Thai sites close together and near to Imus and the tightly clustered Malaysian sites of Penang and Serdang. Quezon City and Lampung were both suggested to be greatly diverged from the other study sites and from each other (Figure 4). Hierarchical AMOVA tests that grouped populations based on *post hoc* MDS plot clusters identified 30.33% of the variation among groups (*P* < 0.05).

**Figure 5:**
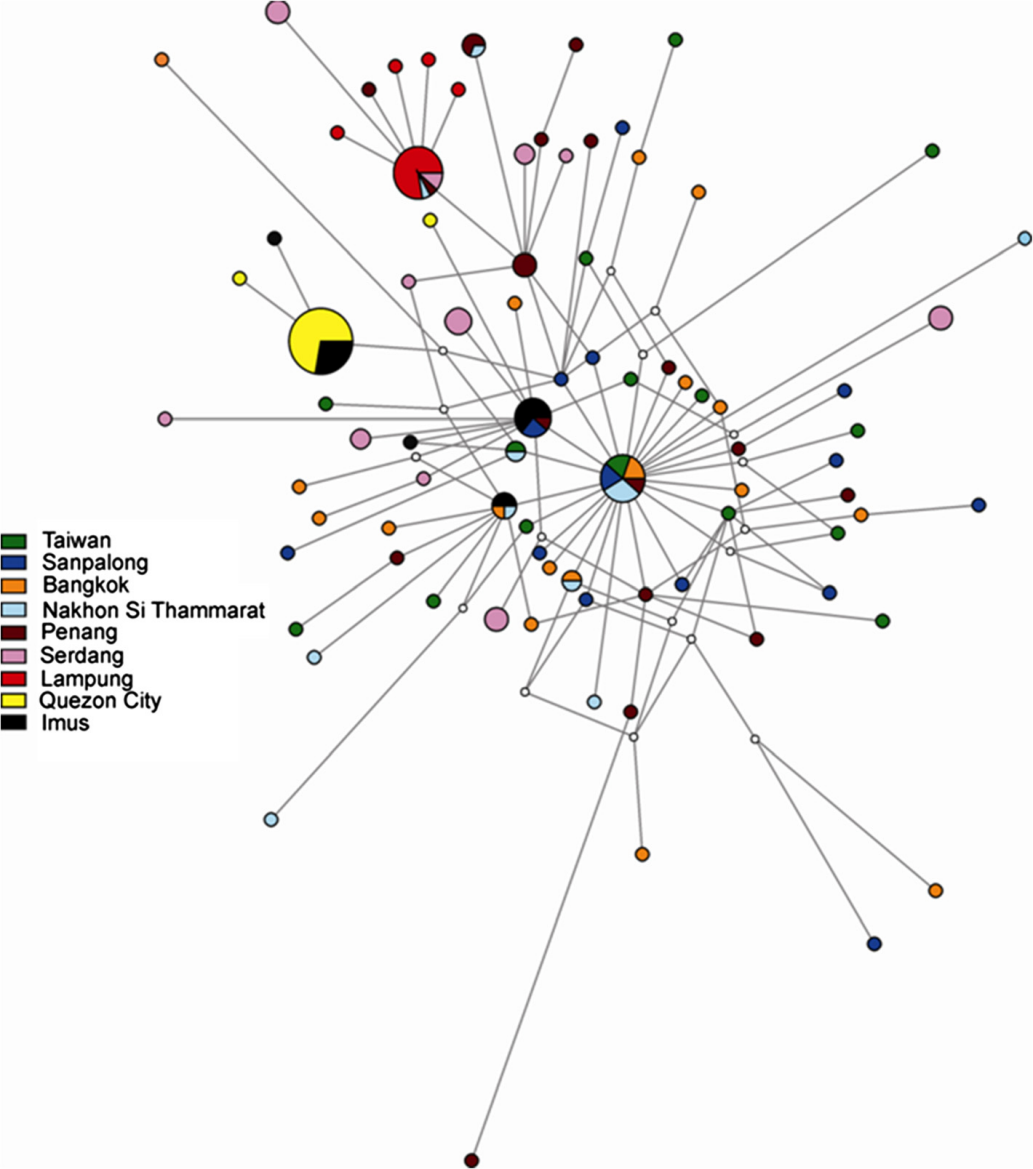
**Haplotype network of* B. dorsalis s.l. *collected in southeast Asia.** COI haplotype network shaded by site, representing 83 haplotypes for *Bactrocera dorsalis* complex flies sampled through southeast Asia. Sizes of nodes and pie segments are proportional to haplotype frequency. Small unshaded circles represent median vectors (roughly equivalent to hypothetical unsampled haplotypes). Length of branches is proportional to number of mutational changes between haplotypes.

The median-joining haplotype network reflected the high observed gene diversity among sampled sites and revealed only a small number of unsampled haplotypes. While unlikely to greatly affect interpretation of the network in the current study, such missing haplotypes can obscure real patterns and so must be considered while interpreting networks. The inferred network did not show any distinct patterns between the haplotypes and their geographical distribution (and inferred taxonomic identity); haplotypes from a given site were generally distributed across the network (Figure 5). This pattern was particularly true for Taiwanese, Thai and Peninsula Malaysian sites, in that there was no evidence for distinct clusters of haplotypes from these sites. Nevertheless, some patterns were forthcoming: haplotypes from Lampung formed a starburst cluster connected to haplotypes from Malaysia; while those from Quezon City and Imus were mixed but seemed to form two separate starburst-like clusters that were connected to Thai haplotypes. Taiwanese and Thai haplotypes appeared to be more central to the network with a distinct central starburst radiation, whilst Malay, Sumatran and Philippine haplotypes were somewhat more derived. Taken together, this would be consistent with a single migration event into Lampung from Malaysia and two migrations into the Philippines from Thailand.

Tests of population expansion using Fu’s *F*_*S*_ were negative and statistically significant across the entire dataset (*F*_*S*_ = −7.133, *P* = 0.019). Individually, six of the nine sites possessed significant estimates of *F*_*S*_ (see Additional file 1: Table S3). The mismatch distribution for the total dataset was unimodal (r = 0.0328, *R*_*2*_ = 0.0219 – see Additional file 1: Figure 2a), suggesting a strong signature of historical population expansion. This was supported by the Bayesian GMRF Skyride plot (see Additional file 1: Figure 2b), which suggested gradual expansion of populations since the mid- to late-Pleistocene (300 - 600kya).

**Figure 6:**
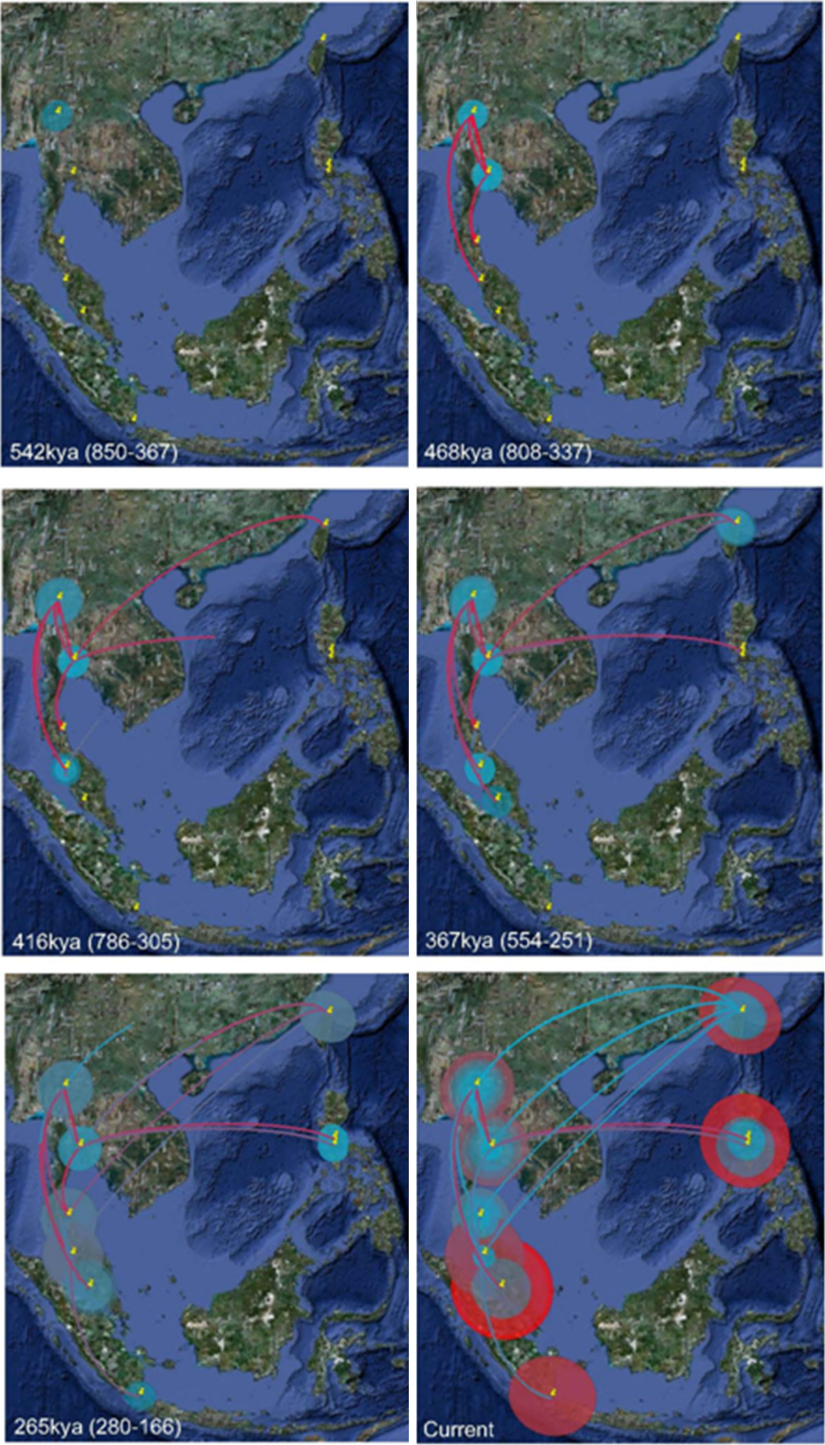
**Hypothesised historical migration paths of* B. dorsalis s.l. *in southeast Asia.** Snapshot images from the output of BEAST discrete phylogeographic analysis showing spread of * B. dorsalis s.l. * haplotypes among populations in a spatial context. Lines drawn between sample sites infer historical migration events. Size of circles represents inferred historical population size. The colour of the lines and circles is indicative of inferred age: red is older, blue is younger. Ages of each snapshot are from the analysis that used a molecular rate of 1.5%/my and values in parentheses are from the runs using the lowest and highest rates, respectively.

Patterns of historical migration among sites revealed by discrete phylogeographic analysis in BEAST support inferences based on median-joining haplotypic relationships (Figure 6). Northern Thailand (San Pa Tong) was supported as the root location across multiple independent runs, with the root-height of the current dataset estimated at approximately 540kya (850 – 367kya). The first migration events inferred were between northern Thailand sites, prior to colonisation of peninsular Malaysia around 470kya (808 – 337kya). Several cycles of immigration and emigration have apparently occurred between Taiwan and mainland Asia beginning around 420kya (786 – 205kya). Two migration events into the Philippines from Thailand are inferred to have occurred between 270-370kya (550 – 166kya). A single colonisation event into Sumatra from Malaysia was supported around 270kya (280 – 166kya).

## Discussion

The pattern of spatial structure resolved for both genetic and geometric morphometric datasets does not correlate with the current taxonomic designations of *B. dorsalis s.s.*, *B. papayae* and *B. philippinensis*. Rather, it is consistent with the hypothesis that these three taxa represent a single species that is widely distributed throughout southeast Asia. Further, our data infer that *B. dorsalis s.l.* has undergone several periods of range expansion throughout its history in southeast Asia. The genetic and geometric morphometric datasets are broadly congruent regarding the biogeographical history of the taxon, albeit with some differences. Based on our sampled range, *B. dorsalis s.l.* originated in northern Thailand and has undergone a gradual range expansion southward along the Thai/Malay peninsula. There followed migration into Sumatra and eastward to the Philippines, although whether that occurred sequentially or concurrently remains unclear. We recovered no evidence of historical movement between the relatively geographically close (~350 km apart) islands of Taiwan and the Philippines.

By broadening the sample range across wider geographic distributions, it becomes possible to re-evaluate relationships within groups of organisms that contain taxa for whose biological identities are unresolved. Such a scenario has recently been highlighted in the southeast Asian region for *Anopheles* mosquitoes, whereby a Philippine species (*A. limosus* King) was redefined as a population of the widespread southeast Asian species, *A. vagus* Dönitz [58]. Similarly, biogeographical population-level studies of *B. dorsalis s.l.* have until now been limited to southern China and northern Asia, the presumed Asian range of *B. dorsalis s.s.*. However, the incorporation of samples of *B. papayae* and *B. philippinensis* with *B. dorsalis s.s.* without *a priori* species boundary assumptions allows for a more extensive biogeographical analysis throughout southeast Asia.

Based on the two independent datasets presented here (shape and mtDNA) there is strong evidence that *B. dorsalis s.l.* had a northern southeast Asian origin, a finding congruent with previous studies [16,22], and that it has subsequently dispersed further southwards into southeast Asia. The movement of flies from northern Thailand appears to have commenced some 540 thousand years ago, reaching the modern Philippines approximately 360 thousand years ago and Sumatra 265 thousand years ago. The COI data suggests the flies may have dispersed in multiple directions, with the correlation of genetic distance against Euclidian distance slighter stronger than the correlation of genetic difference against ‘non-Euclidian’ distance (Figure 3e & f). While there is insufficient difference between these tests to support one hypothesis over the other, the estimated arrival of flies into the Philippines approximately 100,000 years before they arrived in Sumatra (Figure 6) implies that there was not a simple, unidirectional dispersal down Thailand, across Indonesia and up into the Philippines. It may be that flies from (current) central Thailand simultaneously moved both eastward and southward across Sundaland, with the eventual Philippine populations tracking along the eastern (inside) edge of Sundaland (see Figure 1 for Sundaland geography), independent of the populations spreading south into Malaysia and the Indonesian Archipelago. In stark contrast, it is difficult to interpret the wing shape analysis in any way except as a continuous, unidirectional anti-clockwise spread around the South China Sea (Figure 3d). We must emphasise that while results here indicate movement of flies between Thailand and Taiwan began approximately 416kya (Figure 6), this is almost certainly an analytical artifact resulting from our lack of intermittent sample sites in southern mainland China and neighbouring regions (e.g. Vietnam).

While it is possible that the conclusions provided by one methodology are more accurate than the other (i.e. COI *versus* shape data), we believe the two data sets reveal different aspects of the same story. The COI data is resolving older historical gene flow, whereas the limited evidence available (see below) suggests that the shape data may be detecting subtle differences resulting from gene flow in more recent history. Thus it may be that initial colonisation of southeast Asia by *B. dorsalis s.l.* involved dispersal in multiple directions across the exposed Sundaland shelf, while contemporary fly movement is restricted to the current land-arc surrounding the South China Sea, resulting in the extraordinarily strong relationship seen between wing shape and ‘non-Euclidian’ geographic distance. Wing shape variation for these species (and also *B. carambolae*) has previously been examined using historical collection material, with the aim of determining if shape variation could be used as a species discriminatory character [54]. While results from that study demonstrated differences, species from that study were *a priori* defined prior to analysis (unlike here) and, by the nature of the discriminate analysis used, strongly biased results into finding differences. Despite this, a high degree of similarity among taxa (including between *B. dorsalis s.s.* and *B. papayae*) was still identified and it was argued that such variation may be more indicative of that at the intra- rather than inter-specific level, with additional information (such as presented here) required to resolve the true biological relationships as ‘*wing shape information is not, in isolation, an argument to confirm or refute species limits*’ [54]. There are few examples where both geometric morphometric shape data and genetic information have been applied simultaneously to address questions of population structure. In the absence of direct genetic tests, the ability to detect wing shape differences in individuals of the same species subjected to different larval environments [53], or from different sample sites [54], is indirect evidence of the potential of shape data to detect subtle, more recent changes. Other researchers have gone some way towards comparing between shape and genetic data, such as in a study of cranial shape variation in South American *Ctenomys* rodents, which was compared with previously published mtDNA and microsatellite information [55]. While neither genetic nor morphometric data revealed strong population structuring, cranial shape data was nevertheless shown to be as sensitive as molecular data [55]. Less common are direct comparisons between shape and molecular information using the same individuals, however an analysis of wing shape variation *versus* microsatellite data of *Glossina palpalis gambiensis* Vanderplank individuals collected from Burkina Faso, Africa, represents an impressive example of the power of such studies [51]. In this instance the geometric morphometric shape data detected population structuring not evident in the genetic analysis. As COI (used in our study) is not suited to contemporary gene flow as were the microsatellites used for the *G. palpalis gambiensis* study [51], we recommend the future application of microsatellite data for *B. dorsalis s.l.* studies. Regardless of the outcome of future work, however, the differences in the biogeographic stories told by the independent data sets in this paper reinforces the value of using multiple tools in studies of biogeographically complex regions.

We are unable to explain the relatively large divergence in both wing shape and haplotype variation between the two Philippine sites of Quezon City and Imus. Our analysis of mtDNA implies the occurrence of two historical migration events from Thailand, however these two locations are only 24 km apart and we have no reason to believe that the flies used here represent two distinct populations. We suspect that the observed difference may be driven by a haplotype that is shared between Imus, San Pa Tong and Penang. This haplotype is located centrally in the network, several mutational steps from the other more common cluster of Philippine haplotypes and is connected to other haplotypes sampled from mainland sites, along with a further singleton from Imus. Whilst we cannot be sure, we argue that this pattern may be representative of two separate migration events into the Philippines, and the starburst-like pattern of haplotype relationships that characterises the two clusters of Philippine haplotypes, along with low genetic diversity at these two sites, appears to support this scenario. Nevertheless, there are also some haplotypes shared between Quezon City and Imus and the results of the analyses may simply be the consequence of low sample size, especially for Imus (n = 13). Such uncertainty is exacerbated by the relatively large difference in wing shape between the two *B. philippinensis* samples which may represent some form of secondary contact between them and *B. dorsalis s.s.* or *B. papayae*, and while we have not explicitly considered human-mediated movement as a factor influencing observed patterns, it cannot be discounted and also warrants further attention. We therefore recommend this area be re-sampled before asserting hypotheses regarding multiple migrations events into the Philippines. Despite this however, *B. philippinensis* is extremely closely related to *B. dorsalis s.s.* and *B. papayae*, and while specific dispersal pathways remain unclear we believe the patterns observed concur with those expected under an intra-specific hypothesis for these three taxa.

The treatment of these three taxa as a single biological species poses considerable taxonomic implications. While extensive past efforts have been directed toward identifying consistent diagnostic markers for *B. dorsalis s.s., B. papayae* and *B. philippinensis*, this result is yet to be achieved. The question therefore remains: do such diagnostic markers exist but we are looking in the wrong places? Or rather has the original taxonomy been incorrect inasmuch as *B. papayae* and *B. philippinensis* should not be erected to species status but rather considered as populations of *B. dorsalis s.s.*? In light of our results, we believe the latter more likely. If *B. papayae* and *B. philippinensis* are treated as conspecific with *B. dorsalis s.s.* then searching for diagnostic markers to resolve among these ‘species’ is no longer necessary; with the only diagnostic requirement being the discrimination of *B. dorsalis s.s.* from other closely related *B. dorsalis* species complex flies for which morphological or molecular markers often already exist (such as for *B. carambolae*). Notwithstanding this, we recognise the necessity to treat our current study as one line of evidence towards what must be part of a broader integrative taxonomic resolution.

## Conclusions

The three currently defined species of *B. dorsalis s.s.B. papayae* and *B. philippinensis* display genetic and morphometric variation congruent with the hypothesis that they represent the same biological species. Moreover, our data supports a northern southeast Asian origin for *B. dorsalis s.l.* (in accordance with previous studies), with dispersal directed southwards and eastwards into the Malay archipelago and the islands of the Philippines over the last 500,000 years. This historical movement was likely facilitated during periods of lower sea level and the exposure of the vast Sundaland shelf, but gene flow has since been more restricted between mainland southeast Asia and the island chains, possibly due to sea level rises forming geographic barriers, such as that hypothesised for the Isthmus of Kra on the Thai/Malay peninsula and the current seaways between the islands of the Indonesian archipelago. As only the second study to concurrently use genetic and geometric morphometric data, but seeing the same synergies between the approaches as recorded in the first study [51], we support the further use of independent, fine scale morphological information (such as shape analysis) in parallel with genetic data as a means of more completely assessing population structure for biogeographical and related studies. Finally, a reappraisal of the taxonomic relationships among these highly pestiferous tephritid species poses considerable implications for basic research, pest management, quarantine practices and international trade. In recognising the limitations of this study, particularly the potential for further sampling and the use of other independent markers*,* we endorse the need for further studies across other disciplines (e.g. behavioural and cytogenetic) to further clarify the biological relationships among these taxa before any formal taxonomic changes are made.

## Methods

### Study sites & sample collection

Adult male flies were collected from nine sites across southeast Asia; one site in China (Taiwan), three in Thailand, two in peninsular Malaysia, one in Indonesia and two in the Philippines (Figure 1; Table 1). Samples of *B. dorsalis s.s*., *B. papayae* and *B. philippinensis* were collected between May 2009 and December 2010. Flies were collected using methyl eugenol/insecticide baited hanging traps containing propylene glycol as a preserving agent for all sites except Serdang Malaysia. As lures only attract males, females were not incorporated in the analysis. Samples from Serdang (taxonomically confirmed as *B. papayae* by R.A.I. Drew) were reared from *Musa acuminata x balbisiana* hybrids, vars. Mas, Berangan and Lemak bananas collected in November 2010. All samples other than those collected from Serdang were shipped to Queensland University of Technology (QUT), Brisbane Australia, for morphological identification (MKS) and processing; Serdang flies were reared from infested fruit at the UN/FAO International Atomic Energy Agency Agriculture and Biotechnology Laboratories, Seibersdorf Austria. Sample sizes from each site were unknown until the completion of sampling and logistical constraints prevented revisiting sample sites. Flies were identified based on descriptions in Drew & Hancock (1994). Three legs were removed (fore, mid and hind) for genetic analysis and one wing (usually the right) for geometric morphometric shape analysis. There was not a complete 1:1 correlation between material used for genetic and shape analysis due to difficulties in amplifying the COI gene for some wing samples and some flies used for genetic analysis had damaged wings. Nevertheless over 90% of the material examined here had both COI sequence data and wing shape data successfully used. Voucher samples are held at QUT.

### Geometric morphometric analyses

**Figure 7:**
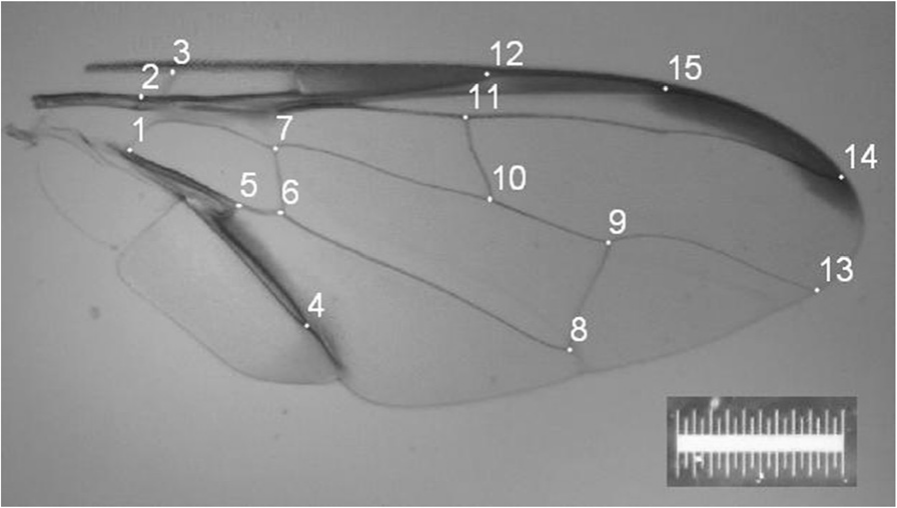
**Right wing of* B. dorsalis s.s. *** Right-hand wing of * Bactrocera dorsalis s.s. * showing the fifteen landmarks used to generate geometric morphometric shape data. Scale = 1 mm.

One wing from each fly was removed for slide mounting, image capture and analysis. Usually the right wing was dissected; however in cases where the right wing was damaged the left was used instead (~10% of instances; approximately evenly distributed across samples). Wings were slide mounted using DPX mounting agent and air-dried prior to image capture using an AnMo Dino-Eye microscope eye-piece camera (model # AM423B) mounted into a Leica MZ6 stereo-microscope. Wing landmark selection (Figure 7) and digitisation followed that undertaken in previous work [54].

The size of each wing was assessed as ‘centroid size’, an isometric estimator of size calculated as the square root of the summed distances of each landmark from the centre of the landmark configuration; and was calculated using the computer program MORPHOLOGIKA v2.5 [59]. One-way analysis of variance followed by the Tukey *post hoc* test was applied to *a priori* groups based on sample location in order to determine significant differences (α = 0.05) among sites with respect to wing size.

Raw landmark coordinate data were imported into the computer program MORPHOJ v1.02E [60] for shape analysis. Data were first subjected to Procrustes superimposition to remove all but shape variation [49]. Multivariate regression of the dependant wing-shape variable against centroid size (independent variable) was conducted to assess the effect of wing size on wing shape (i.e. allometry) [54,61]. The statistical significance of this regression was tested by permutation tests (10,000 replicates) against the null hypothesis of independence. To correct for allometric contribution towards shape variation, subsequent analyses were undertaken using the residual components as determined from the regression of shape on centroid size.

Samples were *a priori* assigned to one of nine sample location groups (as for centroid size analysis), from which subsequent canonical variates analysis (CVA) was applied to determine relative differences in wing shape among groups. Significant differences were determined via permutation tests (1000 permutation rounds) for Mahalanobis distances among groups.

We regressed geographic distance (km) against Mahalanobis distances as calculated from CVA to test for ‘Isolation by Distance’ (IBD) effects [62]. Geographic distance was calculated in one of two ways to test the hypothesis that population variation was structured around the South China Sea by: 1) Euclidean geographic distance and 2) ‘non-Euclidean’ geographic distance. Euclidean distances represented the shortest possible geographic distance between pairs of sample locations with no prior biogeographic assumptions; whereas ‘non-Euclidean’ distance was measured as the sum of all respective distances between sample sites extending through the peninsula, into the archipelago and up to the Philippines (see Figure 3 a & b). For example, the Euclidean distance between Taipei and Quezon City is 1,155 km (the shortest distance possible), whereas the ‘non Euclidean’ distance is the sum of distances between all sample sites from Taiwan, across to the mainland, down the peninsula, into the archipelago and up to the Philippines (7,586 km). The ‘non-Euclidean’ distance measure was used as it closely approximated geographic distances between sample sites for when sea levels were lower (i.e. when more of the ‘Sundaland’ land mass was exposed) and therefore represents our hypothetical pathway for historic land-restricted dispersal by *B. dorsalis s.l*. The strength of the association for either approach was determined by linear regression analysis using the program SPSS v17.0.

### Genetic analyses

Leg material for genetic analysis was sent to the Elizabeth Macarthur Agricultural Institute (EMAI), New South Wales Australia, for DNA extraction. DNA was extracted from each fly using the Qiagen DNeasy Blood and Tissue kit according to the manufacturer’s instructions which was then subaliquoted into a master stock and stored at −20 °C as two working stock solutions. One working stock was sent to Lincoln University, Christchurch New Zealand, for sequencing of a 642 bp fragment of the mitochondrial cytochrome *c* oxidase subunit I (COI) gene.

Polymerase chain reactions were undertaken with forward FolA and reverse FolB primers [63] using either (1) the Expand High Fidelity (HiFi) PCR System (Roche Diagnostics GmbH, Mannheim, Germany) with 1 mM MgCl2, primers at 60 ng each, 0.2 mM dNTPs, 1× PCR buffer, 0.525 U enzyme mixture and 0.7 μL DNA in a total volume of 10 μL or (2) GoTaq® Green Master Mix (Promega, Madison, USA) with primers at 250 ng each and 0.5 μL of DNA template in a total volume of 20 μL. Thermocycling conditions were 94 °C for 2 min, then 40 cycles of 94 °C for 15 s, 50 °C for 30 s and 72 °C for 45 s, followed by 7 min at 72 °C. We used PCR boost (Biomatrica, San Diego, USA) as a substitute for water in cases where samples failed to produce enough PCR product for sequencing using Expand High Fidelity (HiFi) PCR System. All PCR products were checked in 1% agarose gels containing SYBR SafeTM DNA Gel Stain (Invitrogen, Carlsbad, USA) in 0.5× TBE buffer. Both directions of PCR products were sequenced at the Lincoln University Bio-Protection Research Centre, using primers FolA and FolB and ABI Big Dye (ABI, Foster City, USA) technology on ABI PRISM 3130xl Genetic Analyzer (ABI, Foster City, USA) according to the manufacturer’s recommendations. COI sequences are available under the GenBank Accession Numbers JX099580 - JX099755.

COI sequences were aligned using BioEdit Version 7.0.5 [64] and checked by eye for discrepancies. Tests for sequence saturation were conducted by calculating the mean ratio of transitions to transversions in MEGA Version 4.0 [65]. Tajima’s D tests of neutrality were estimated for the total dataset and for each individual population using 1000 coalescent simulations in Arlequin Version 3.11 [66] to determine if sequences were evolving neutrally. Gene diversity and the population parameter, θπ, were calculated using Arlequin to estimate genetic diversity within sites. A haplotype network was constructed using the median-joining method followed by maximum parsimony post-processing in Network Version 4.6.0.0 [67]. Supporting information for the exclusion of *B. carambolae* is presented as a haplotype network including *B. carambolae* which reveals it to share no haplotypes with the three target species (*n =* 20; 13 unique haplotypes; Additional file 1: Figure S3)*.* These 20 specimens of *B. carambolae* were field collected from Serdang, Malaysia (native distribution) and Paramaribo, Suriname (invasive distribution).

Various methods for partitioning genetic variation within and among sites were implemented. Conventional among-site ΦST indices (*P* < 0.05) incorporating the Tamura-Nei model of evolution [68] were estimated in ARLEQUIN to explore the level of connectivity among sites. We used hierarchical analyses of molecular variance (AMOVA [69]) computed in ARLEQUIN using ΦST estimates to test hypotheses of *a priori* site groupings. Statistical significance for these methods was obtained through 1000 random permutations. Clustering of sites based on relative ΦST estimates was performed using multidimensional scaling in accordance with [70] and using the ALSCAL analysis in the PASW Statistics Version 18 software package (formerly SPSS). Populations are converted to points in a two-dimensional space, with the linear distances between points proportional to the relative ΦST estimates among populations. Similar to wing-shape data, hypotheses of IBD were assessed by linear regression analysis between geographical distance (Euclidean and ‘non-Euclidean’) and genetic distance among groups (ΦST).

We tested hypotheses of post-isolation population expansion by estimating Fu’s FS [71] for the total dataset and for each site individually in ARLEQUIN (obtaining statistical support via 1000 coalescent simulations) and by plotting the mismatch distribution of pairwise differences and their frequency in DnaSP Version 5.0 [72]. We also plotted relative population size changes over time using Bayesian Gaussian Markov Random Field (GMRF) Skyride Plots [73] in BEAST Version 1.5.3 [74]. Due to the lack of useful fossil calibrations for this analysis, we attempted to account for molecular rate uncertainty in two ways. First, we used an uncorrelated lognormal relaxed molecular clock model to allow rates to vary along branches. Second, we ran three sets of analyses using the fastest (2.28%/my – [75]) and slowest (0.9%/my – [76]) insect mitochondrial substitution rates, as well as a standard invertebrate mitochondrial divergence rate of 1.5%/my [77] as the rough median. We employed a Hasegawa-Kishino-Yano (HKY) substitution model and a time-aware prior on the smoothing of the scaled effective population size. Two runs of 30 million generations each were implemented for the three analyses and log and tree files were combined in LogCombiner Version 1.5.3 [74] to produce GMRF Skyride Plots.

To investigate the geographical spread of haplotypes among the study sites through time we implemented the discrete phylogeographical analysis in BEAST [78]. We used a Bayesian stochastic search variable selection (BSSVS) procedure and incorporated a relaxed lognormal molecular clock, Bayesian GMRF Skyride demographic model and HKY substitution model as described above. We ran three sets of analysis using the three molecular rates described above to provide an age range for inferred migration events. Two runs of 30 million generations each were performed for each analysis and the resulting geotree files were combined and annotated with location states in TREEANNOTATOR Version 1.5.3 [74]. The annotated tree was then converted to a keyhole markup language (KML) file using SPREAD [79], a program that converts the output of discrete phylogeographic analysis from BEAST into a ‘kml file’ prior to visualisation in Google Earth. Convergence of runs – and thus support for the inferred ages of migration events – was achieved by ensuring estimated sample sizes (ESS) for the ‘geotreelikelihood’ prior were greater than 200 in the combined log file of each analysis.

## Competing interests

The authors declare that they have no competing interests.

## Authors’ contributions

MKS coordinated and undertook specimen collection, carried out wing-shape analysis and wrote the manuscript. MNK constructed the molecular dataset, undertook genetic analysis and wrote the manuscript. ARC contributed to writing the manuscript. TAC and AE extracted DNA and undertook sequencing analysis. AC undertook sequencing analysis. This work was undertaken as part of a larger project developed by ARC, DH, KFA, SLC and MKS. All authors read and approved the final manuscript.

## Supplementary Material

Additional file 1 Additional file contains three supplementary figures and four tables. Click here for file
