# Supplementary material for: Population structure of Bactrocera dorsalis s.s., B. papayae and B. philippinensis (Diptera: Tephritidae) in southeast Asia: evidence for a single species hypothesis using mitochondrial DNA and wing-shape data
Source: BMC Evol Biol. 2012 Jul 30;12:130. doi: 10.1186/1471-2148-12-130 (PMC3458884; doi:10.1186/1471-2148-12-130)
Supplement: Additional file 1 — Additional file contains three supplementary figures and four tables. [file 1471-2148-12-130-S1.pdf]

Additional data for:

Population structure of *Bactrocera dorsalis s.s.*, *B. papayae* and *B. philippinensis* (Diptera: Tephritidae) in southeast Asia: evidence for a single species hypothesis using mitochondrial DNA and wing-shape data.

Mark K. Schutze, Matthew N. Krosch, Karen F. Armstrong, Toni A. Chapman, Anna Englezou, Anastasija Chomič, Stephen L. Cameron, Deborah Hailstones and Anthony R. Clarke

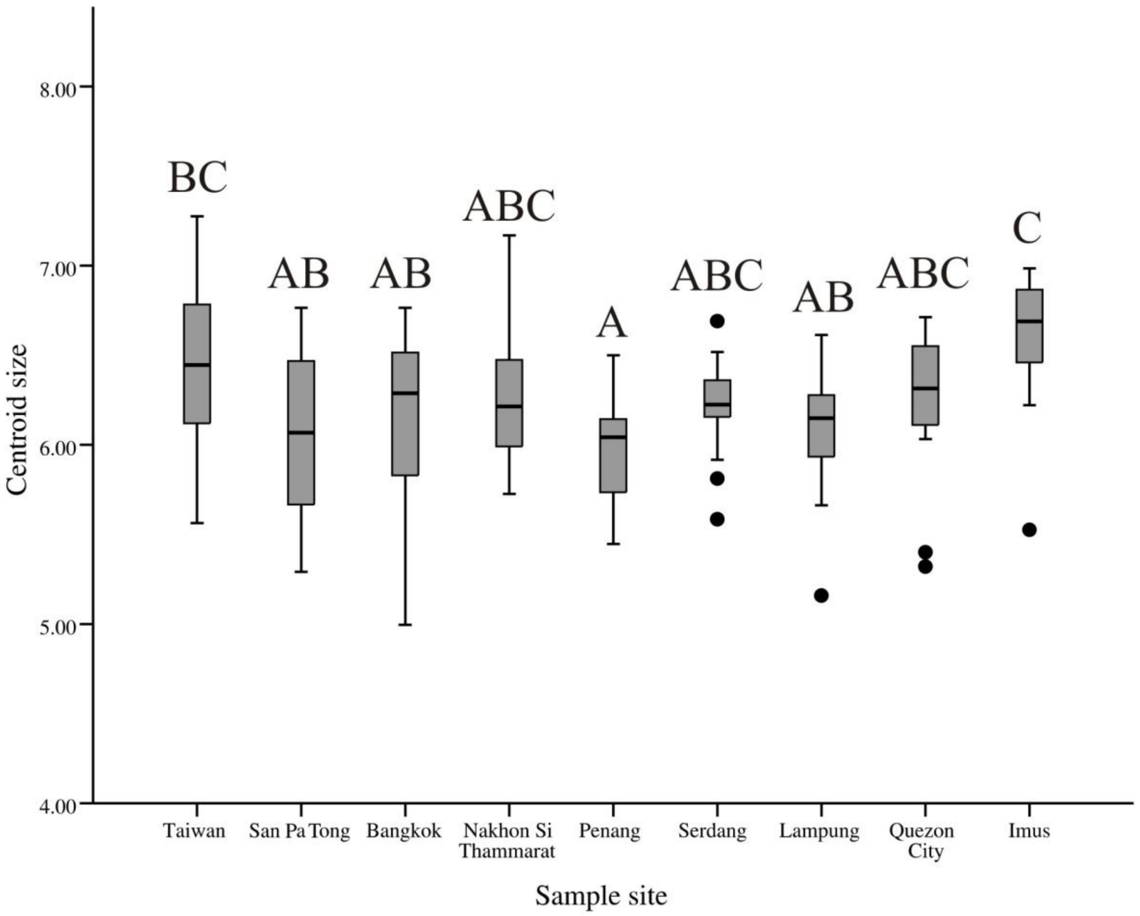

Supporting figure 1. Centroid sizes for individuals from nine sample sites of *Bactrocera dorsalis s.l.* showing median observation (black bar), lower and upper quartiles (box), minimum and maximum observations (whiskers), and outliers (dots). Sample sites sharing the same letter are not significantly different ($P > 0.05$) based on ANOVA with Tukey *post hoc* test.

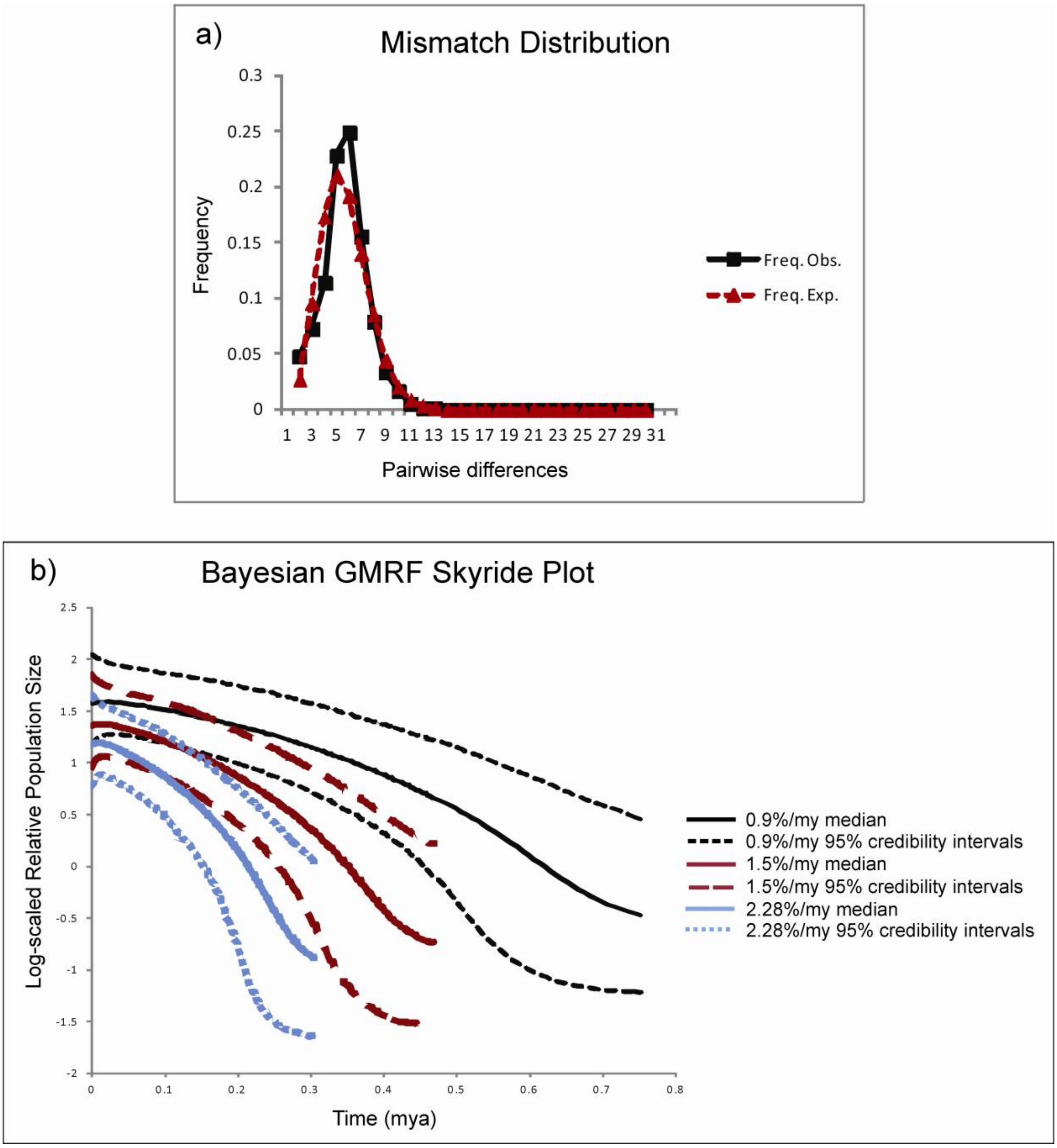

Supporting figure 2. (a) Mismatch distribution plot of COI pairwise difference frequencies ($\theta_{initial}$ = 0.000, θ_{final} = 1000, τ = 3.648); (b) Bayesian GMRF Skyride plot inferring population size change over time. The *y*-axis shows relative log-scaled population size as a function of effective population size and generation time and the *x*-axis shows time in millions of years from the present. Solid lines indicate the median Skyride estimate and dashed lines show upper and lower 95% credibility intervals.

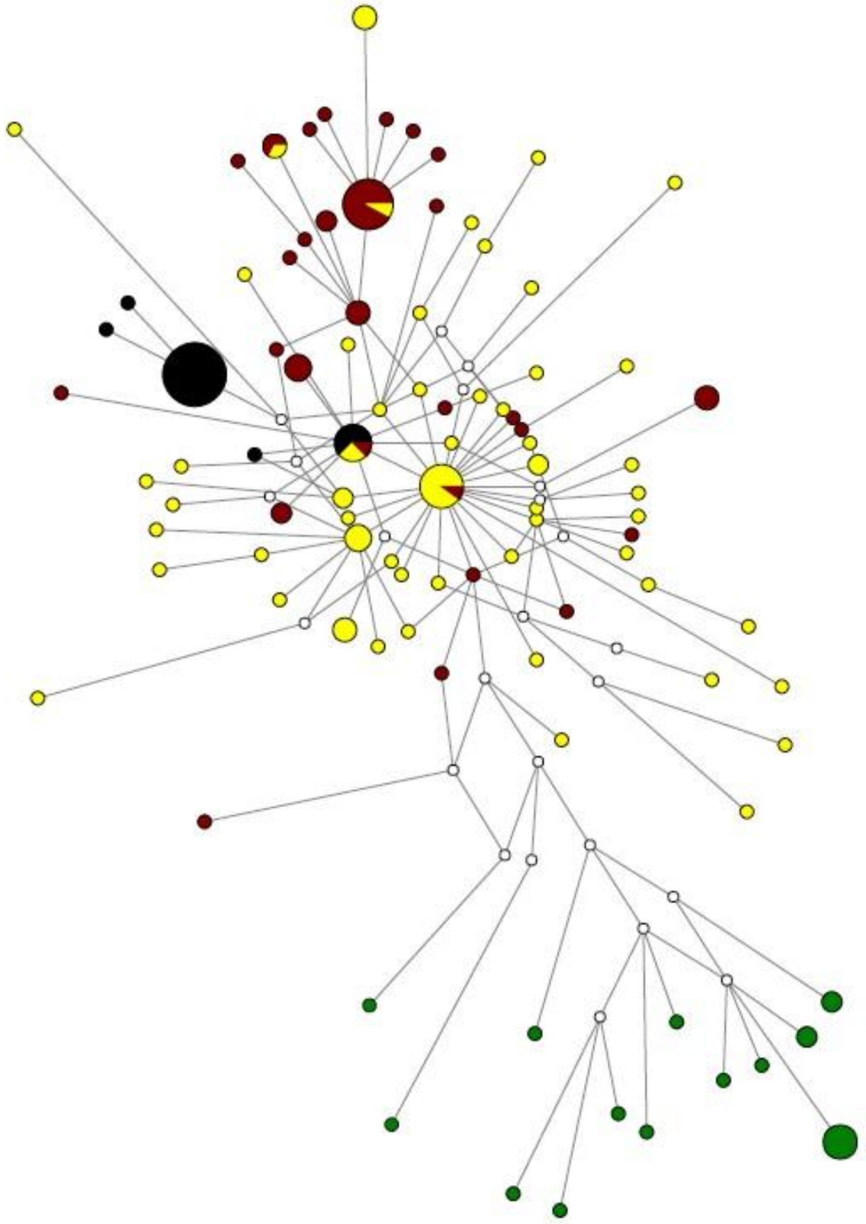

Supporting figure 3. COI haplotype network shaded by species, representing 96 haplotypes for *Bactrocera dorsalis* complex flies sampled through southeast Asia. Yellow = *B. dorsalis s.s.*; maroon = *B. papayae*; black = *B. philippinensis*; green = *B. carambolae*. Size of nodes and pie segments are proportional to haplotype frequency. Small unshaded circles represent median vectors (roughly equivalent to hypothetical unsampled haplotypes). Length of branches is proportional to number of mutational changes between haplotypes.

Supporting table 1. Eight canonical variates (CV) produced from CVA on nine groups of *Bactrocera dorsalis s.l.*

CV	Eigenvalue	% Variance	Cumulative %
1	4.01	55.05	55.05
2	1.09	14.93	69.98
3	0.70	9.65	79.63
4	0.68	9.31	88.93
5	0.34	4.70	93.64
6	0.21	2.90	96.54
7	0.15	2.10	98.64
8	0.10	1.36	100.00

Supporting table 2. Relative wing shape distances of *Bactrocera dorsalis s.l.* male flies among groups collected from nine geographic sites in Southeast Asia. Above diagonal = Procrustes distances; below diagonal = Mahalanobis distances (from CVA computed from Procrustes shape data corrected for allometry). Bold values statistically significant (α = 0.05).

	Taiwan	Sanpalong	Bangkok	Nakhon Si Thammarat (NST)	Penang	Serdang	Lampung	Quezon City	Imus
Taiwan	-	**0.0114**	**0.0135**	**0.0125**	**0.0132**	**0.0161**	**0.0188**	**0.0307**	**0.0247**
San Pa Tong	**3.2593**	-	0.0044	0.0087	0.0076	**0.0118**	**0.0141**	**0.0259**	**0.0178**
Bangkok	**3.6012**	1.6047	-	0.0086	0.0075	0.0103	**0.0139**	**0.0229**	**0.0154**
NST	**3.7567**	**2.3913**	**2.1253**	-	0.0077	0.0097	**0.0130**	**0.0229**	**0.0192**
Penang	**3.7815**	**2.5887**	**2.3918**	**2.3018**	-	0.0075	0.0093	**0.0244**	**0.0171**
Serdang	**3.9698**	**2.888**	**2.4999**	**2.3595**	**2.4356**	-	0.0078	**0.0206**	**0.0145**
Lampung	**4.5662**	**3.3163**	**3.2421**	**3.1022**	**2.122**	**2.4593**	-	**0.0246**	**0.0158**
Quezon City	**6.9312**	**5.8487**	**5.2753**	**5.2948**	**5.1750**	**4.5220**	**5.3327**	-	**0.0184**
Imus	**6.2350**	**4.6661**	**4.2638**	**4.7364**	**4.2271**	**3.7848**	**3.8841**	**3.8269**	-

Supporting table 3. Population genetic summary statistics. Values in bold are statistically significant ($\alpha = 0.05$).

Sample site	Sample size	θ_π	Gene Diversity	Tajima's D	Tajima's D p-value	Fu's F_S	Fu's F_S p-value
Taiwan	19	3.42690	0.9825 +/- 0.0223	-1.95625	**0.007**	-12.3915	**0.000**
San Pa Tong	19	3.30994	0.9825 +/- 0.0223	-1.93455	**0.021**	-12.7125	**0.000**
Bangkok	19	4.16374	0.9942 +/- 0.0193	-1.89615	**0.016**	-16.1392	**0.000**
Nakhon Si Thammarat	12	3.81818	0.9545 +/- 0.0569	-1.72052	**0.038**	-4.73496	**0.005**
Penang	20	3.81579	0.9789 +/- 0.0245	-1.47379	0.051	-12.8921	**0.000**
Serdang	22	4.63203	0.9264 +/- 0.0287	-0.57881	0.314	-1.6083	0.233
Lampung	15	0.53333	0.4762 +/- 0.1545	-1.81594	**0.015**	-3.22826	**0.000**
Quezon City	17	0.11765	0.1176 +/- 0.1012	-1.16387	0.141	-0.74844	0.106
Imus	13	2.28205	0.7436 +/- 0.0866	1.48433	0.946	0.25899	0.555

Supporting table 4. Pairwise among site Φ_{ST} estimates of differentiation below diagonal. Values in bold are statistically significant (α = 0.05). Euclidean geographic distances (km) above diagonal with 'non-Euclidean' geographic distances in parentheses.

	Taiwan	Sanpalong	Bangkok	Nakhon Si Thammarat	Penang	Serdang	Lampung	Quezon City	Imus
Taiwan	-	2439 (2439)	2520 (3000)	2943 (3608)	3138 (3938)	3240 (4254)	3823 (5313)	1156 (8139)	1179 (8116)
San Pa Tong	-0.01158	-	561 (561)	1140 (1169)	1470 (1499)	1764 (1815)	2801 (2874)	2397 (5700)	2394 (5676)
Bangkok	-0.00442	-0.01972	-	607 (607)	931 (938)	1211 (1253)	2241 (2312)	2204 (5138)	2196 (6977)
Nakhon Si Thammarat	0.00198	0.01417	-0.00003	-	330 (330)	634 (646)	1691 (1705)	2401 (4531)	2387 (4508)
Penang	**0.09434**	**0.08972**	**0.08579**	**0.05208**	-	316 (316)	1373 (1374)	2484 (4201)	2467 (4176)
Serdang	**0.09123**	**0.0774**	**0.07511**	**0.08423**	**0.06161**	-	1059 (1059)	2482 (3885)	2463 (3862)
Lampung	**0.55828**	**0.56744**	**0.51405**	**0.53647**	**0.34674**	**0.37555**	-	2826 (2826)	2803 (2803)
Quezon City	**0.57614**	**0.60432**	**0.54375**	**0.61876**	**0.5428**	**0.51772**	**0.92804**	-	24 (24)
Imus	**0.20357**	**0.21352**	**0.1837**	**0.23135**	**0.23995**	**0.18166**	**0.69318**	**0.40894**	**-**
